# Antipyretic Use in Noncritically Ill Patients With Fever: A Review

**DOI:** 10.7759/cureus.51943

**Published:** 2024-01-09

**Authors:** Khawar Tariq Mehmood, Shahad Al-Baldawi, Gabriel Zúñiga Salazar, Diego Zúñiga, Sneha Balasubramanian

**Affiliations:** 1 Internal Medicine, Aster Hospital Br of Aster DM Healthcare FZC, Dubai, ARE; 2 Department of Rheumatology, Al-Yarmouk Teaching Hospital, Baghdad, IRQ; 3 Medicine, Universidad Católica de Santiago de Guayaquil, Guayaquil, ECU; 4 Internal Medicine, Madras Medical College, Chennai, IND

**Keywords:** noncritical, toxicity, nsaids, acetaminophen, antipyretics, pyrexia, fever

## Abstract

Antipyretics are one of the most frequently used agents in medicine. Numerous pharmacological agents, such as acetaminophen, non-steroidal anti-inflammatory agents (NSAIDs), salicylates, and selective cyclooxygenase 2 (COX-2) inhibitors, and nonpharmacological treatment modalities, such as tepid sponging and cooling blankets, are available for temperature reduction. There is a scarcity of definitive clinical guidelines on the choice of various agents in noncritically ill febrile patients. Our review examined the various modalities available for antipyresis and compared their safety and efficacy. The rationale for the choice of a particular pharmacological agent and route of administration were scrutinized. Our review also envisaged the perceived beneficial effects of antipyretics against the harmful side effects, including the evaluation of morbidity or mortality advantage conferred by antipyretics. The various toxicities associated with these agents were also highlighted.

## Introduction and background

Fever or pyrexia is caused by an increased hypothalamic thermoregulatory setpoint due to several infectious and non-infectious causes. The various inciting agents cause a release of endogenous or exogenous pyrogens (fever-producing agents) that act on the neurological system to instigate a prostaglandin-mediated alteration in the temperature setpoint [1]. An increase in body temperature offers numerous physiological advantages in times of stress or infection possibly due to changes in the host immune system [2]. The same adaptive response can also prove detrimental by increasing the body’s metabolic demand, oxygen consumption, minute ventilation, and contributing to adverse neurological outcomes [3-5]. Several pharmacological and non-pharmacological treatment modalities are available for mitigating fever.

Drugs, such as paracetamol, nonsteroidal anti-inflammatory drugs (NSAIDs), and cyclooxygenase 2 (COX-2) inhibitors, and physical therapies, such as cooling blankets and immersion, are commonly used in febrile individuals to achieve temperature reduction [6-8]. Despite fever being a ubiquitous symptom, the evidence surrounding the selection of an appropriate antipyretic regimen, dosing, route of administration, and drug choice is limited [9]. Some studies even suggest that antipyretic use in infectious etiologies has detrimental outcomes. This may be due to the loss of microbial suppressive effects of fever [10,11]. This premise is supported by the theoretical risk of relative immunosuppression caused by normothermia [12]. Furthermore, antipyretics can also have prominent hemodynamic side effects and can contribute to renal and hepatic dysfunction [13-15]. Selective toxicities of the available antipyretics may limit their use in specific patients. The data on the usage of antipyretics for controlling temperature are limited when considering noncritical patients. There are no clear-cut guidelines or recommendations specifying the choice of a particular agent, the route of administration, or data on comparative safety and efficacy.

This literature review aimed to analyze the various available modalities of temperature control and their relative safety and efficacy. The review also investigated factors that may help direct the selection of a particular agent. Finally, the advantages and disadvantages of fever control and toxicities of the antipyretics were evaluated.

## Review

Pathophysiological basis of fever

**Figure 1:**
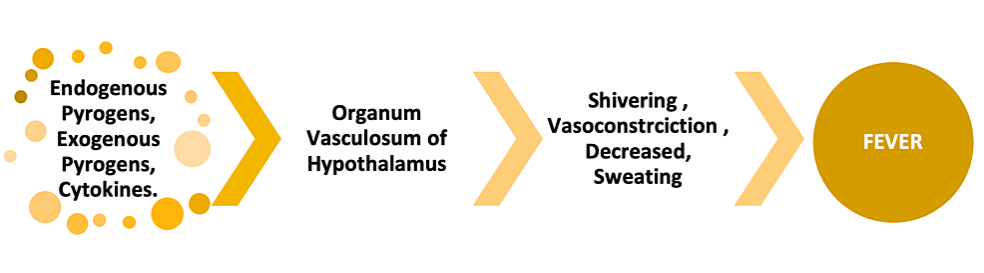
Pathophysiology of fever Image credits: Khawar Tariq Mehmood

Fever is an adaptive response that results in increased body temperature secondary to an alteration in the physiological temperature setpoint in the hypothalamus [16]. This alteration is caused by increased prostaglandin synthesis in the organum vasculosum of lamina terminalis by numerous inflammatory mediators. Some of these mediators include cytokines, such as tumor necrosis factor (TNF), interleukin (IL)-6, and IL-1, which can be themselves produced in response to exogenous pyrogens. Furthermore, there is evidence that local cytokine production and other neurohormonal mechanisms may be responsible for alteration in the hypothalamic setpoint [17]. Alteration of the setpoint then leads to bodily responses that raise the core body temperature. These responses, like many other coordinated biological processes, are coordinated in the hypothalamus [18]. The basic steps involved in the generation of febrile response are illustrated below (Figure 1).

Pyrexia has been shown to have an inhibitory effect on microorganism proliferation and amplifies the endogenous immunological response. This amplification is seen throughout the innate and adaptive immune response. Increased neutrophilic recruitment, release, and activity, enhanced natural killer (NK) cell cytolytic activity, stimulation of phagocytic activity of macrophages and dendritic cells, and increased lymphocytic trafficking are some of the ways fever modulates the immune response [2,19]. These pyrexia-induced alterations aid in controlling and eliminating the offending infectious agent. Although seemingly advantageous from an evolutionary point of view, fever has numerous deleterious consequences on the human body. Increased metabolic demand, increased minute ventilation, and cardiovascular and neurological stresses are some of the harmful effects of fever [20,21]. Despite its ubiquitous nature, the management of pyrexia remains controversial. Fever tends to be a source of discomfort both for the patient and the clinician.

Choice of Antipyretic Agent in Febrile Patients

Alleviation of fever has been a common therapeutic target for hundreds of years [22]. The therapeutic rationale behind this is to reduce patient discomfort and mitigate the effects of increased metabolic demand and risk of hypoxic neurological injury that can be caused by fever [3,4]. Numerous pharmacological and non-pharmacological treatment options are available for antipyresis [23]. Common pharmacological agents include acetaminophen and NSAIDs, such as ibuprofen, salicylates, and novel COX-2 inhibitors, such as celecoxib and rofecoxib. The most likely mechanism behind the various antipyretics is the inhibition of prostaglandin E2 synthesis in the hypothalamus [24]. 

A comprehensive analysis of data on the relative potencies of various antipyretic drugs is not possible due to differing formulations, routes of administration, and measures of efficacy reported in various studies [21]. Nevertheless, several studies in children have found that oral ibuprofen is a more potent antipyretic than oral acetaminophen though the difference is small [25-27]. Another study also showed that other NSAIDs, such as nimesulide and ketoprofen, were also useful antiinflammatory agents in pediatric patients [28]. In children, therefore, oral ibuprofen can be considered initially for fever control.

NSAIDs can also be used effectively for fever management in adults. A study conducted by Michie et al. demonstrated that pretreatment with ibuprofen blunted the symptoms resulting from an increase in cytokines, such as tumor necrosis alpha in endotoxin-challenged volunteers [29]. Another randomized, double-blind, placebo-controlled trial conducted by Bernard et al. highlighted the effectiveness of ibuprofen in reducing the systemic effects of fever. This study conducted between October 1989 and March 1995 on 455 patients who presented with sepsis compared the effect of intravenous ibuprofen (10 mg/kg per dose, maximum dose 800 mg, given every eight hours for six doses) with that of placebo. The study found a significant reduction in urinary levels of prostacyclin and thromboxane A2, along with reductions in temperature, heart rate, oxygen consumption, and lactate levels in patients treated with ibuprofen. However, no significant reduction in the development of shock, acute respiratory distress syndrome, or mortality was reported [30]. 

Another study conducted by Vargas et al. in endotoxin-challenged volunteers compared the efficacy of oral acetaminophen and intramuscular ketorolac. This double-blind, double-dummy, parallel study showed that increasing doses of ketorolac are associated with a higher antipyretic effect and comparative efficacy was seen between 30 mg intramuscular ketorolac and 650 mg oral acetaminophen [31]. This comparative efficacy of the various antipyretics was further highlighted in the double-blind trial conducted by Reiner et al., which compared the efficacy of nimesulide with that of diclofenac and placebo. It was seen that nimesulide suppositories were as effective as diclofenac suppositories in the reduction of fever and mitigation of objective signs of fever (pulse and blood pressure), and both were superior to placebo [32]. 

The data suggest that various antipyretic agents are effective in temperature reduction, and thus the choice of agent used should be determined by the individual patient profile. The several antipyretic groups have varying toxicity profile that plays a crucial role in the selection of a particular agent. 

Salicylates, such as aspirin, have been linked with the development of Reye’s syndrome in children [33]. This rare but catastrophic childhood disorder results from the inhibition of hepatic mitochondrial oxidative phosphorylation and subsequent development of hepatic failure and encephalopathy [34]. NSAIDs have a myriad of adverse effects involving almost any organ system (Table 1).

**Table 1:** Common side effects of nonsteroidal anti-inflammatory drugs Table credits: Khawar Tariq Mehmood

Organ system affected	Toxicities
Gastrointestinal system	Peptic ulcer, esophagitis, small and large bowel erosions
Renal	Acute renal failure, chronic renal failure, acute interstitial nephritis, nephrotic syndrome, electrolyte abnormalities
Cardiovascular	Worsening of hypertension, angina and exacerbation of heart failure
Hepatic	Hepatic failure
Hematological	Thrombocytopenia, hemolytic anemia, aplastic anemia
Neurological	Headache, drowsiness, confusion, aseptic meningitis
Respiratory	Nasal polyposis, exacerbation of asthma
Integumentary	Rash

Renal and gastrointestinal toxicity from NSAIDs results from the inhibition of COX isoforms [35]. Agents with nonselective inhibition of COX enzymes have been found to cause greater gastrointestinal toxicities than agents that selectively inhibit COX-2 enzyme isoforms [36]. Other important factors, such as age of more than 60 years, presence of previous gastrointestinal disorder, prolonged duration of NSAID use, and concomitant intake of other agents with gastrointestinal toxicity, such as corticosteroids, also increase the risk of gastrointestinal toxicity [37]. Drugs, such as rofecoxib, selectively inhibit COX-2 isoforms, which are associated with a higher likelihood of cardiovascular adverse effects, such as myocardial infarction and stroke possibly by promoting a more thrombogenic environment [38]. NSAIDs are one of the most common agents associated with renal toxicity with effects, ranging from interstitial nephritis to acute or chronic renal failure [39]. Individuals with preexisting renal disease and those using nephrotoxic agents are at an increased risk of developing renal dysfunction [40]. 

Unlike NSAIDs, acetaminophen has little activity against COX enzymes and thus has minimal gastrointestinal and renal toxicity [36]. It is however metabolized to a potentially hepatotoxic intermediate known as N-acetyl-p-benzoquinoneimine (NAPQI) [41]. The risk of toxicity increases in individuals with depleted glutathione reserves, e.g., chronic ethanol ingestion and starvation. [42]. Thus, the choice of antipyretic agent depends on the patient's demographic, preexisting medical conditions, and concurrent medication usage. It is of utmost importance to administer the selected antipyretic for the shortest duration and at the lowest effective dose to limit systemic toxicity.

Pros and Cons of Fever

**Figure 2:**
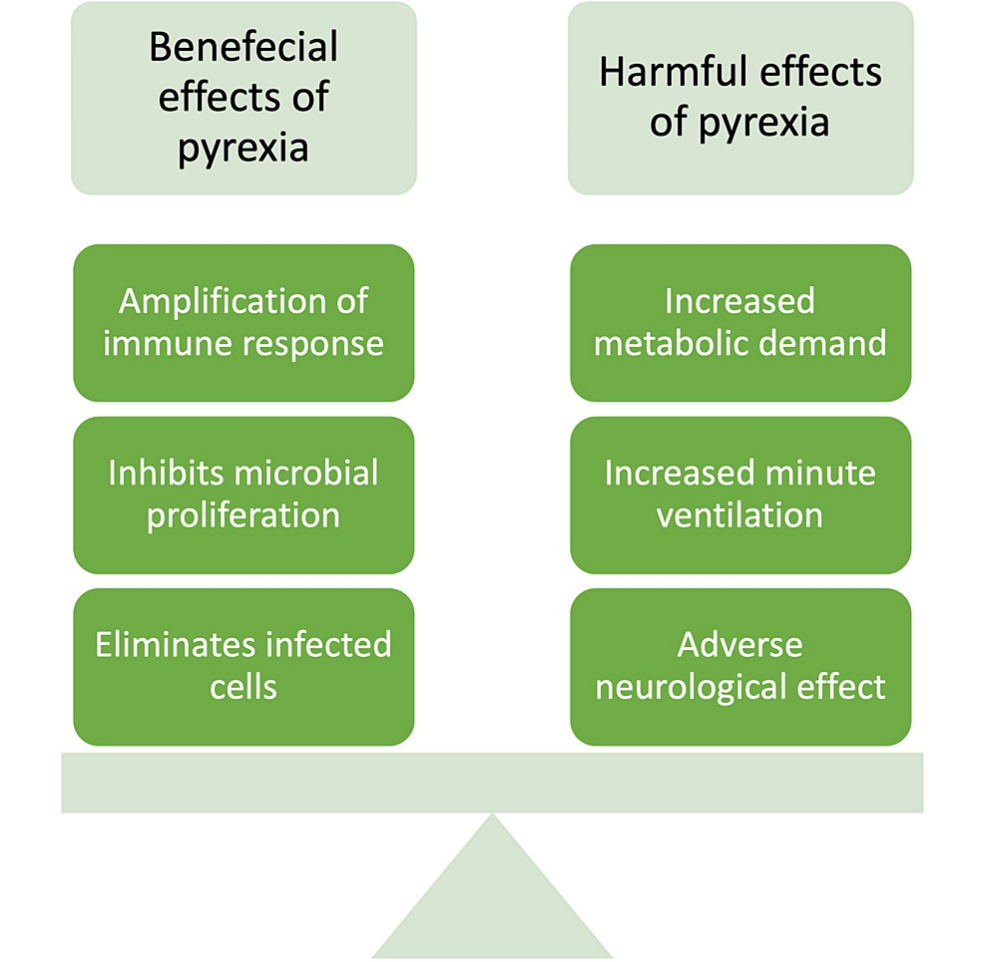
Weighing the beneficial and harmful effects of pyrexia Image credits: Khawar Tariq Mehmood

Whether control of the body’s physiological response of the body to inflammation offers any quantifiable benefit is debatable. Like any treatment modality, the risks and benefits of fever and its control should be sensibly considered (Figure 2).

**Figure 3:**
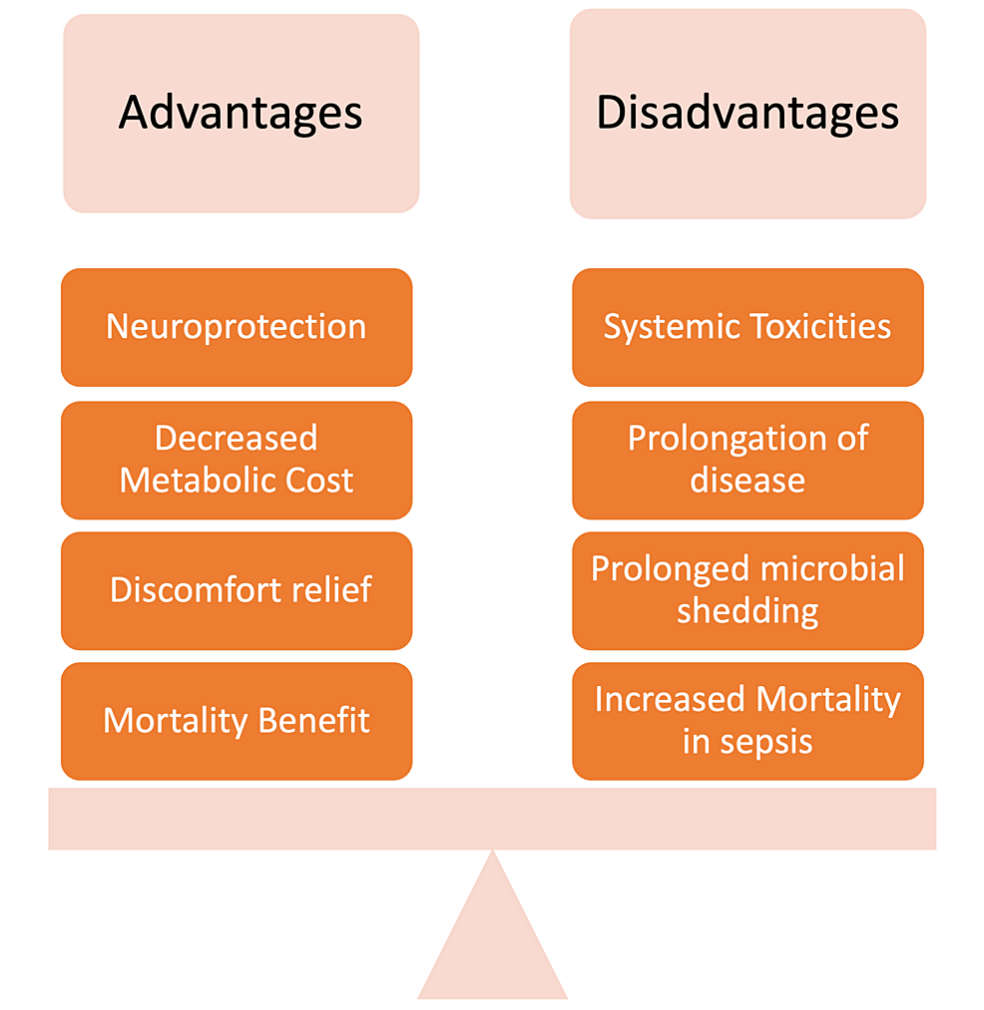
Some of the advantages and disadvantages of antipyretic use for controlling fever. Image credits: Khawar Tariq Mehmood

The use of antipyretics for the control of fever has been ongoing for ages [43]. The pros and cons of antipyretic use should also be considered before instituting treatment (Figure 3).

Suggested Advantages of Fever Control

Pyrexia can cause significant discomfort in a febrile patient and relieving patient discomfort is an important reason for prescribing antipyretics. Improving patient well-being is an important therapeutic rationale in febrile individuals. Children who are irritable and pyretic show prompt improvement after the administration of an antipyretic agent [44]. This effect was demonstrated in a randomized clinical trial conducted by Ipp et al., in which 383 infants aged two to six months and 70 children aged 18 months were studied for frequency and severity of adverse reactions following administration of diphtheria-pertussis-tetanus toxoids-polio vaccine. The study showed that acetaminophen-treated infants between the ages of two to six months had a lower incidence of local and systemic effects, a lower incidence of fever, and a reduction in behavioral changes compared to placebo. However, this effect was not seen in infants aged 18 months. It was concluded that acetaminophen use reduced the frequency of common adverse effects at the time of administration of primary vaccination with diphtheria-pertussis-tetanus toxoids-polio [45] (Table 2).

**Table 2:** Hypothesized beneficial effects of antipyretic agents.

References	Design	Population	Selection criteria	Sample size	Conclusion
Chiappini et al. (2023) [46]	Single-center prospective observational study	Children	Febrile children admitted to a pediatric emergency department	172	Paracetamol is associated with a reduction in body temperature and discomfort relief.
Oborilová et al. (2002) [47]	Non-randomized open-label pilot study	-	Predominantly hemato-oncological patients with axillary temperature at least 38°C	-	Antipyretics provide a useful therapeutic option for the alleviation of discomfort.
Reichenberg et al. (2001) [48]	Double-blind, crossover study	Healthy male volunteers	-	20	Endotoxin exposure is associated with a rise in core body temperature (0.5°C) depressed verbal and non-verbal memory functions, increased anxiety, and depressed mood.
Bernard et al. (1997) [30]	Randomized, double-blind, placebo-controlled trial	-	Patients with sepsis	455	Ibuprofen does not reduce mortality or decrease the development of shock or respiratory distress.
Manthous et al. (1995) [3]	-	-	Febrile, critically ill, mechanically ventilated patients	12	Cooling patients is associated with a reduction in oxygen consumption, carbon dioxide production, and caloric expenditure.
Ipp et al. (1987) [45]	Randomized clinical trial	Children	Children aged 2-6 months and 18 months receiving diphtheria-pertussis-tetanus toxoids-polio vaccine	519	Acetaminophen administered at the time of primary vaccination with diphtheria-pertussis-tetanus toxoids-polio reduces the frequency and severity of common adverse reactions.
Beisel et al. (1974) [49]	-	-	Volunteers with experimentally induced sandfly fever	-	Reduction in work performance did not occur in individuals who received symptomatic therapy.

Another single-center prospective observational study conducted by Chiappini et al. in 172 febrile children who were admitted to a pediatric emergency department showed the beneficial effects of acetaminophen in alleviating fever and discomfort. This study analyzed the effect of acetaminophen on fever and discomfort (defined using a semiquantitative Likert scale) and found significant reductions in both body temperature and levels of discomfort at 60 minutes when compared to baseline in children treated with oral paracetamol [46] (Table 2). 

This beneficial effect on discomfort relief was also reported by Oborilová et al. in their non-randomized open-label pilot study comparing the effects of three antipyretics agents in various mainly hemato-oncological patients. A total of 254 episodes of fever (axillary temperature of at least 38°C) were treated with either diclofenac (75 mg, brief intravenous (IV) infusion) or metamizol (2500 mg or 1000 mg, brief IV infusion) or propacetamol (2000 mg or 1000 mg, slow IV injection or brief IV infusion). Changes in axillary temperature, improvement in discomfort, and adverse effects were recorded. It was observed that all antipyretics were associated with a reduction in temperature and improvement in patient discomfort (87% of patients declared improvement in comfort). However, efficacy, tolerability, and occurrences of adverse events differed between the groups. It was concluded that antipyretics provide a useful therapeutic option for the alleviation of discomfort [47] (Table 2).

Pyrexia is associated with increased metabolic strain on the body via increased cardio-respiratory rate and oxygen consumption. Lowering temperature has been suggested as an important therapeutic target to counteract the metabolic strain on the body. This effect was highlighted in a study conducted by Manthous et al., which analyzed the effect of cooling on oxygen consumption in febrile critically ill patients. This study showed that cooling resulted in a statistically significant reduction in oxygen consumption, carbon dioxide production, and energy expenditure in 12 febrile, mechanically ventilated patients when the temperature was reduced from 39.4 +/- 0.8°C to 37.0 +/- 0.5°C [3] (Table 2).

High fever has been linked to cognitive dysfunction and brain damage. This adverse effect on neurological function was demonstrated in a double-blind, crossover study conducted by Reichenberg et al. in 20 healthy male volunteers exposed to intravenous injection of *Salmonella abortus equi *endotoxin (0.8 ng/kg) while compared to saline. The study showed that endotoxin exposure was associated with a rise in core body temperature (0.5°C) and depressed verbal and non-verbal memory functions. Endotoxin exposure was also associated with a transient but significant increase in anxiety levels and depressed mood [48] (Table 2). Unfortunately, this study did not examine the ability of antipyretics to prevent these neuropsychological disturbances. Another study conducted by Beisel et al. did show a reduction in illness-related decrements in work performance in volunteers with experimentally induced sandfly fever. The beneficial effect was even observed when fever and other symptoms were not completely relieved [49] (Table 2). The effect of fever in patients with neurological conditions, such as stroke, is more well-studied. A meta-analysis conducted by Hajat et al. suggested that pyrexia is associated with increased morbidity and mortality in patients with acute stroke [50]. When considering the numerous above-mentioned benefits, it has been postulated that fever reduction may provide some degree of survival benefit. This hypothesis was tested by Bernard et al. in a randomized, double-blind, placebo-controlled trial conducted in 455 septic patients between October 1989 to March 1995 comparing the effects of ibuprofen to that of placebo. The research concluded that ibuprofen administration was associated with a reduction of prostaglandin and thromboxane synthesis and subsequently with reduced fever, oxygen consumption, and lactate production. However, it did not prevent the development of shock or respiratory distress or offer any mortality benefit [30] (Table 2).

Suggested Disadvantages of Fever Reduction

Antipyretics like any other treatment are not completely free from adverse effects. Apart from the myriad of adverse effects mentioned above fever reduction can result in the production of certain less than favorable effects. An example is the interruption of constitutively produced prostaglandins by NSAIDs. This blockade in the production of endogenous mediators can have detrimental effects on various organ systems. In a study done by Friedman et al., indomethacin administration to patients with coronary artery disease was associated with an increase in coronary vascular resistance and a decrease in coronary blood flow. This effect was likely related to the decreased synthesis of vasodilatory prostaglandins [51] (Table 3). Hence, a cautious approach is needed when prescribing these agents in patients with underlying coronary artery disease. One major drawback of fever reduction is the theoretical risk of blunting an organism's natural immune response. Mitigation of this protective, natural response thus has been postulated to prolong illness. Evidence supporting this was seen in the randomized, double-blind, placebo-controlled trial conducted by Doran et al., which studied the effects of acetaminophen on childhood varicella. The study enrolled children between the ages of one and 12 years who had chickenpox and randomized them to receive either acetaminophen (n = 37) or placebo (n = 31), and symptoms, such as pruritis, appetite, activity, and overall condition were measured along with time to eruption of last vesicle, time to scabbing of all lesions, and time to total healing. One child was withdrawn and three did not complete the study. It was seen that the time to total scabbing and pruritis were better in placebo when compared with the active treatment. Thus, it was concluded that acetaminophen plays no significant role in the alleviation of symptoms in children with varicella and may prolong illness duration [52] (Table 3).

**Table 3:** Disadvantages of fever control.

References	Design	Population	Selection criteria	Sample size	Conclusion
Friedman et al. (1981) [51]	-	-	Patients with coronary artery disease	9	Indomethacin infusion was associated with increased coronary vascular resistance and decreased coronary blood flow.
Doran et al. (1989) [52]	Randomized, double-blind, placebo-controlled trial	Children	Children between the ages of one and 12 years who had chickenpox	72	Acetaminophen plays no significant role in the alleviation of symptoms in children with varicella and may prolong illness duration.
Graham et al. (1990) [53]	Double-blind, placebo-controlled trial	-	Healthy volunteers challenged intranasally with rhinovirus type 2	60	Aspirin and acetaminophen were associated with statistically significant suppression of serum-neutralizing immunoglobulins and non-statistically significant trend toward longer illness.
Plaisance et al. (2000) [54]	Retrospective observational study	Volunteers	Volunteers with experimentally induced influenza A, S. sonnei, R. rickettsii infections	120	Prolongation of illness with use of antipyretics in Influenza A and Shigella sonnei infections but not with R. rickettsiiinfections.
Lee et al. (2012) [55]	Prospective observational study	Adult	Adult critically ill patients (without neurological injury) requiring >48 hours intensive care	1425	Non-steroidal anti-inflammatory or acetaminophen use was independently associated with increased 28-day mortality in septic patients but not in non-septic patients.
Ye et al. (2017) [56]	-	-	Critically ill patients with sepsis requiring mechanical ventilation	8,711	Antipyretic therapy is associated with increased risk of mortality in septic patients requiring mechanical ventilation.

This tendency of fever control in prolonging the duration of self-limited illness is also seen in other studies. In a double-blind, placebo-controlled trial conducted by Graham et al., the effect of commonly used over-the-counter analgesic/antipyretics on virus shedding, immunological response, and clinical status of 60 healthy volunteers challenged intranasally with rhinovirus type 2. The participants were randomized to receive aspirin, acetaminophen, ibuprofen, or placebo. Fifty-six were infected and showed evidence of viral shedding. Subsequently, viral shedding, immunoglobulin levels, symptoms and signs, and white blood cell counts were carefully monitored. It was surprisingly seen that aspirin and acetaminophen were associated with statistically significant suppression of serum-neutralizing immunoglobulins and increased nasal signs and symptoms. No statistically significant difference was seen in viral shedding among the groups, but there was a trend toward longer viral shedding with acetaminophen and aspirin use [53] (Table 3). 

A similar prolongation of illness associated with the use of antipyretics was also seen in a retrospective observational study conducted by Plaisance et al., which evaluated the effects of antipyretics on experimental influenza A, *Shigella sonnei*, and *Rickettsia rickettsii* infections. The participants comprised of individuals with experimentally induced influenza A (n = 54), *S. sonnei* (n = 45), and *R. rickettsii *(n=21) infections. Acetaminophen or aspirin was offered for symptomatic relief. Multivariate analysis revealed prolongation of illness associated with the use of antipyretics in individuals with influenza A and *S. sonnei* infections but not with *R. rickettsii* infections [54] (Table 3).

Some studies even suggest that antipyretics may be harmful in select groups of patients and may be related to increased mortality. A multi-center, prospective observational study conducted by Lee et al. studied the effect of fever and antipyretics on mortality in 1,425 adult critically ill patients (without neurological injury) with and without sepsis. Individuals who required intensive care of at least 48 hours were included in the study. It was found that NSAID or acetaminophen use was independently associated with increased 28-day mortality in septic patients but not in non-septic patients [55] (Table 3).

A similar increase in mortality in septic patients treated with antipyretics was also seen by Ye et al. in critically ill patients with sepsis and requiring mechanical ventilation (n = 8711) [56] (Table 3).

Limitations

Our review was limited to the studies indexed in the PubMed database. Another limitation was the lack of recent randomized controlled trials evaluating the efficacy of antipyretics in noncritically ill patients. These shortcomings are in addition to the inherent limitations of narrative review, such as lack of completeness of literature review, potential bias in interpretation, and objectivity.

## Conclusions

Several pharmacological and nonpharmacological agents are available to combat fever. The numerous pharmacological agents usually have comparative efficacy and tolerability, and the choice of agent usually depends on the patient profile. When oral tolerability is poor or rapid relief is required, parenteral formulation of antipyretic agents should be considered. The patient’s age, comorbid conditions, nutritional status, and concurrent medication use must be considered when prescribing antipyretics. The selective toxicity profile of various pharmacological agents can also guide prescription.

Whether any meaningful benefit is gained from the administration of antipyretics is still questionable. Prompt alleviation of patient discomfort may be an important therapeutic target for both clinicians and patients. Whether pursuing this therapeutic target comes at the cost of relative immunosuppression and prolongation of illness is not known. Antipyretic administration has not been shown to provide any major morbidity or mortality benefit and may cause more harm than good in septic patients. Stroke is an area where temperature control is has shown to produce favorable outcomes. Whether this neuroprotection translates to noncritical patients is an area that needs further clarification. Finally, no agent is universally free from adverse events. All antipyretics can cause serious toxicities though the spectrum of organ involvement varies according to the class of antipyretics used.

Our review shows that the perceived beneficial effects of antipyretics may be overstated and their use excessive. Unnecessary drug administration may be causing more harm than good and restrictive use may reduce healthcare costs and decrease the risk of complications. Antipyretic administration like all medical interventions should be carefully weighted. Their risks and benefits are considered. Further studies are needed to determine if no intervention is an acceptable option for noncritically ill febrile patients.
